# P-1471. Mortality Analysis of Antimicrobial Regimens Selection in *Acinetobacter baumannii* Ventilator-Associated Pneumonia

**DOI:** 10.1093/ofid/ofae631.1641

**Published:** 2025-01-29

**Authors:** Axel Lozano, Paola L Rondan, Jaime T Martínez, Alvaro Taype-Rondan

**Affiliations:** Universidad Nacional Mayor de San Marcos, Lima, Callao, Peru; Universidad Nacional Mayor de San Marcos, Lima, Callao, Peru; Hospital de Lima Este Vitarte / Universidad Nacional Mayor de San Marcos, Lima, Lima, Peru; Universidad San Ignacio de Loyola, Lima, Lima, Peru

## Abstract

**Background:**

Ventilator-associated pneumonia from *Acinetobacter baumannii* is one of the most severe healthcare-associated infections. Its increased antimicrobial resistance presents challenges in treatment and is associated with high mortality rates. The objective of this study was to determine the association between high dose ampicillin-sulbactam and mortality in patients with ventilator-associated pneumonia from *Acinetobacter baumannii* (AB-VAP).

Clinical epidemiological characterization of patients with AB-VAP

**Figure:**
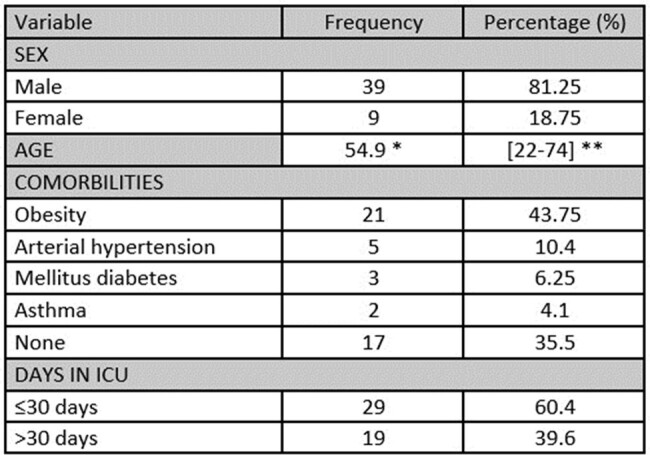

*Median

**Interquartile range

**Methods:**

We performed a retrospective cross-sectional study reviewing electronic medical records of patients from the Intensive Care Unit of a referral hospital for COVID-19 between April 2020 and May 2021.

Characterization according to antibiotic therapy in patients with AB-VAP

**Figure:**
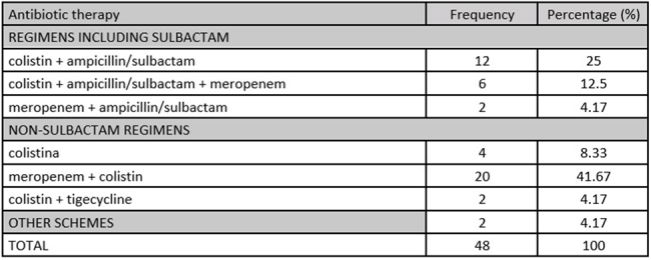

**Results:**

We reviewed a total of 311 medical records. 48 patients with *AB*-VAP were selected according to inclusion and exclusion criteria. The study population was 81.25% male and the median age was 54.9 years [IQR: 22-74]. The most common comorbidities were obesity (43.75%) and hypertension (10.4%). Patients requiring mechanical ventilation for more than 30 days had higher mortality rates compared to patients requiring less than or equal to 30 days (73.68% vs. 44.83%, p=0.049). The most commonly used antibiotic regimens were meropenem plus colistin (41.67%) and colistin plus ampicillin-sulbactam (25.0%). The non-sulbactam antibiotic regimens had a higher 14-day mortality than the sulbactam regimens (60.71% vs 20%, p=0.005). The mortality risk was 2.99 (CI: 1.19 - 7.49, p=0.019) times higher when using regimens without sulbactam, adjusted for sex, SAPS II score and days in the ICU.

Bivariate analysis of antibiotic regimens and mortality in patients with AB-VAP

**Figure:**
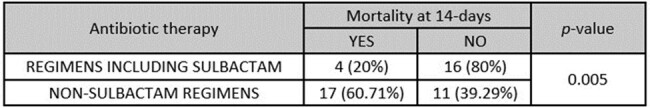

**Conclusion:**

The mortality risk with non-sulbactam antibiotic regimens was 3 times higher compared to sulbactam antibiotic regimens in patients with *AB*-VAP.

Multivariate Poisson Regression Analysis of antibiotic therapy and mortality in patients with AB-VAP

**Figure:**
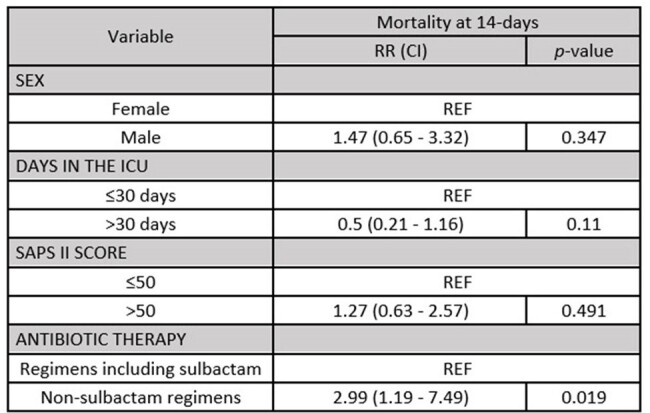

**Disclosures:**

**All Authors**: No reported disclosures

